# A statistical framework for modeling gene expression using chromatin features and application to modENCODE datasets

**DOI:** 10.1186/gb-2011-12-2-r15

**Published:** 2011-02-16

**Authors:** Chao Cheng, Koon-Kiu Yan, Kevin Y Yip, Joel Rozowsky, Roger Alexander, Chong Shou, Mark Gerstein

**Affiliations:** 1 Department of Molecular Biophysics and Biochemistry, Yale University, 260 Whitney Avenue, New Haven, CT 06520, USA; 2 Department of Computer Science and Engineering, The Chinese University of Hong Kong, Rm 1006, Ho Sin-Hang Engineering Bldg, Shatin, New Territories, Hong Kong; 3 Program in Computational Biology and Bioinformatics, Yale University, 260 Whitney Avenue, New Haven, CT 06520, USA; 4 Department of Computer Science, Yale University, PO Box 208285, New Haven, CT 06520, USA

## Abstract

We develop a statistical framework to study the relationship between chromatin features and gene expression. This can be used to predict gene expression of protein coding genes, as well as microRNAs. We demonstrate the prediction in a variety of contexts, focusing particularly on the modENCODE worm datasets. Moreover, our framework reveals the positional contribution around genes (upstream or downstream) of distinct chromatin features to the overall prediction of expression levels.

## Background

In eukaryotes, nuclear chromosomes are organized into chains of nucleosomes, which are in turn composed of octamers of four types of histones wrapped around 147 bp of DNA. Modifications of these core histones are central to many biological processes, including transcriptional regulation [1], replication [2], alternative splicing [3], DNA repair [4], apoptosis [5,6], gene silencing [7], X-chromosome inactivation [8] and carcinogenesis [9,10]. Among them, transcriptional regulation is one of the most important and thereby intensively investigated processes [1,11,12]. Histone modifications have been demonstrated to regulate gene transcription in positive or negative manners depending on the modification site and type [13-18]. For example, a genome-wide map of 18 histone acetylation and 19 histone methylation sites in human T cells indicates that H3K9me2, H3K9me3, H3K27me2, H3K27me3 and H4K20me3 are negatively correlated with gene expression, whereas most other modifications, including all the acetylations, are correlated with gene activation [18,19]. As an extreme case, histone modifications play critical roles in X-chromosome inactivation in females to equalize the expression of X-linked genes to those in male animals [19,20]. Histone modifications are thought to affect transcription through two mechanisms: modifying the accessibility of DNA to transcription factors by altering the local chromatin structure; and providing specific binding surfaces for the recruitment of transcriptional activators and repressors [11,17,21-23].

The large number of possible histone modifications has led to the 'histone code' hypothesis, which states that combinations of different histone modifications specify distinct chromatin states and bring about distinct downstream effects [24-26]. Moreover, one histone modification may influence another by recruiting or activating chromatin-modifying complexes [27]. However, a study in yeast revealed only simple and cumulative functional consequences for combinations of histone H4 acetylation rather than a complicated synergistic histone code [28]. Two other studies, one in yeast and the other in *Drosophila*, also demonstrated that histone modifications are highly correlated with each other and are partially redundant in function [13,17], presumably conferring robustness in relation to epigenetic regulation [29]. Alternatively, the high correlation between histone modifications may have been overestimated as a result of differences in nucleosome density or other unknown biases [29]. So far, knowledge about the effect of histone modifications on transcriptional regulation is still limited, and the degree of complexity of the histone code is far from clear. To further understand the relationship between histone modifications and gene expression, we require a systematic analysis that integrates histone modification maps with other genome-wide datasets.

The model organism encyclopedia of DNA elements (modENCODE) project was launched in 2007 for the purpose of generating a comprehensive annotation of functional elements in the *Caenorhabditis elegans *and *Drosophila melanogaster *genomes [30]. By using recently developed genome-wide experimental techniques such as ChIP-chip, ChIP-seq and RNA-seq [31,32], modENCODE has generated a large amount of data, including gene expression profiles, histone modification profiles, and DNA binding data for transcription factors and histone-modifying proteins. This large compendium of datasets provides an unprecedented opportunity to investigate the relationship between chromatin modifications and transcriptional regulation using an integrative approach.

In this study, we endeavor to construct a general framework for relating chromatin features with gene expression. We apply a multitude of supervised and unsupervised statistical methods to investigate different aspects of gene regulation by chromatin features. Leveraging the rich data generated by the modENCODE project, we use *C. elegans *as a primary model to illustrate our formalism. Nevertheless, we tested the generality of our methods using a variety of species ranging from yeast to human. More specifically, we show that chromatin features can accurately predict the expression levels of genes and collectively account for at least 50% of the variation in gene expression. We also study the importance of individual features, examine the combinatorial effects of chromatin features, and investigate to what extent the histone code hypothesis is valid. By applying the chromatin-based model to predict the expression of coding genes and microRNAs at different developmental stages, we further address the developmental stage specificity of chromatin modifications and suggest that chromatin features regulate transcription of coding genes and microRNAs in a similar fashion.

As more and more genome-wide ChIP-Seq and RNA-Seq data are going to be generated via the modENCODE project and the ENCODE project [2] in the near future, the methods of data integration proposed in this work have various potential applications.

## Results

### Chromatin features show distinct signal patterns around genic regions

**Figure 1:**
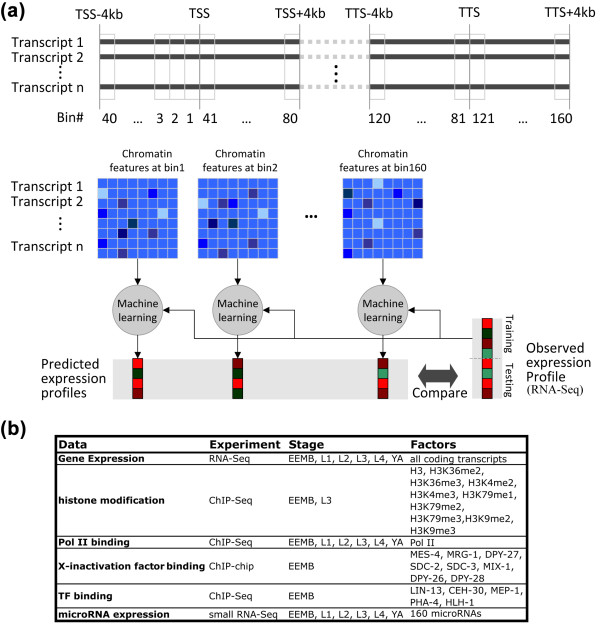
**Schematic diagram of our data binning and supervised analysis**. **(a) **DNA regions around the transcription start site (TSS) and transcription terminal site (TTS) of each transcript were separated into 160 bins of 100 bp in size. Average signal of each chromatin feature was calculated for all transcripts, resulting in a predictor matrix for each bin. These predictor matrices were used to predict expression of transcripts by support vector machine (SVM) or support vector regression (SVR) models. The genome-wide data for chromatin features and gene expression were generated by the modENCODE project using ChIP-chip/ChIP-seq and RNA-seq experiments, respectively. **(b) **A summary of datasets used in our analysis. L, larval; TF, transcription factor; YA, young adult.

**Figure 2:**
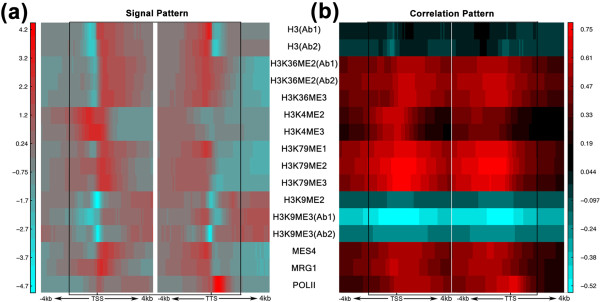
**Chromatin feature patterns**. **(a,b) **Signal pattern (a) and correlation pattern (b) of each chromatin feature in the 160 bins around the TSS and TTS (from 4 kb upstream to 4 kb downstream) of worm transcripts at the EEMB stage. In (a), the signal of each chromatin feature for each bin is averaged across all transcripts. In (b), the Spearman correlation coefficient of each chromatin feature with gene expression levels was calculated for each bin. Ab1 and Ab2 represent experimental results using different antibodies for a chromatin feature. DNA region from 2 kb upstream of the TSS to 2 kb downstream of the TTS is shown in the rectangle.

To systematically study the genome-wide properties of various chromatin features, we collected more than 50 ChIP-chip and ChIP-seq profiles of histone modifications and DNA binding factors in *C. elegans *from the modENCODE project (see Materials and methods). We divided the DNA regions around (± 4 kb) the transcription start site (TSS) and transcription termination site (TTS) of each transcript into small 100-bp bins and calculated the average signal of the chromatin features in each bin. As a result, each bin was assigned a matrix whose elements are the average signals of different features in different transcripts (Figure 1). Figure 2a shows the rich spatial pattern of 16 features in the early embryonic (EEMB) stage, where the signals are averaged over all transcripts. We first observed that the upstream and downstream regions of TSSs and TTSs are clearly distinct. Most chromatin features have higher signals in the transcribed regions (downstream of TSSs and upstream of TTSs). Interestingly, we found that RNA polymerase II (Pol II) has the strongest binding signal in regions right after the TTS, rather than within the transcribed region (Figure 2a). The enriched binding signals right after the TTS may indicate the importance of anti-sense transcription as a regulatory mechanism for gene expression [14,33]. Strong Pol II signal was also observed at regions before the TSS in some other developmental stages (Figure S1 in Additional file 1), which was also reported previously in *C. elegans *by [34], and was thought to be related to the accumulation of TSS-associated RNAs in mouse and human [35,36]. The signal pattern of histone H3 suggests that nucleosomes have lower occupation density in regions around the TSS and TTS than within the transcribed regions. H3K4me2 and H3K4me3 are enriched upstream of the TSS, consistent with their reported role as histone marks for active promoters [14]. On the other hand, signals for H3K9me2 and H3K9me3 are depleted around TSS compared to neighboring regions, which may reflect the low density of nucleosomes around the TSS of genes [28].

### Chromatin features exhibit distinct spatial correlation patterns with gene expression levels

The different chromatin features display distinct spatial patterns. It is thus worthwhile to explore the relationship between these patterns and the level of gene expression. Making use of RNA-seq data obtained from the different stages of *C. elegans*, we quantified the expression level of each gene. For each bin, we then calculated the correlation between the gene expression levels and the average signals of each chromatin feature of the bin. Figure 2b shows the spatial variation of these correlation coefficients around TSSs and TTSs. According to the correlation patterns, there are two main types of chromatin features: ones that are positively correlated with gene expression (such as H3K79me1, H3K79me2 and H3K79me3); and ones that are negatively correlated with gene expression (such as H3K9me2 and H3K9me3). While some features show largely uniform correlations across the 16-kb regions, some others are more variable across the regions. For example, H3K79me2 has a high correlation coefficient (0.65) near the TSS, but rather a low correlation (0.10) downstream of the TTS. It is interesting to observe that the negative features tend to have more uniform spatial patterns while the positive features tend to show greater variation. In addition, for chromatin features such as H3K79me2, although the average signal intensity decreases with distance downstream from the TSS, the correlation between the feature signal and the expression level remains high. This pattern suggests that, while some chromatin features have the strongest average signals only at some highly specific regions, the differences of their signals between genes with low and high expression levels remain strong over much broader regions.

We chose the long window size of 4 kb in order to inspect how fast the signals of the chromatin features fade out as we move away from the TSS and TTS. Indeed, the correlations of some chromatin features (for example, H3K9me3) remain strong a few kilobases away from the TSS and TTS, and the fading could only be observed at the 4-kb boundaries. To make sure that our conclusions are not affected by short genes with some bins having both the identities of being within 4 kb downstream of the TSS and within 4 kb upstream of the TTS, we also did the correlation analysis only on transcripts longer than 8 kb, and found that the correlation patterns are the same (Figure S2 in Additional file 2). Also, as the *C. elegans *genome is quite compact, the region 4 kb upstream of a TSS or downstream of a TTS could be overlapping with another gene. We thus repeated the analysis using transcripts that are at least 4 kb away from any other known transcripts, and again obtained similar correlation patterns (Figure S3 in Additional file 3). Furthermore, analysis based on bins within intergenic regions again resulted in a similar correlation pattern. Therefore, the high correlation of gene expression with feature signal at distant locations does reflect the long-range effects of their regulation, instead of an artifact caused by chromatin structure of the nearby genes.

Furthermore, to assess whether the trends we observed are universal to all developmental stages rather than specific to the EEMB stage, we repeated the analysis in other stages, including late embryo, larval stages and young adult. Although the exact values of correlation coefficients vary across stages, the spatial patterns are consistent in all stages (Figure S4 in Additional file 4). In addition, a large number of genes are associated with multiple transcripts corresponding to different alternative splicing isoforms. In many cases, the overlap between these transcripts is substantial, which might affect the correlation patterns between chromatin features and expression. We thus repeated the correlation analysis using only genes with a single transcript, and obtained the same qualitative results (Figure S5 in Additional file 5).

Among the chromatin features shown in Figure 2, MES-4 and MRG-1 are factors associated with X-chromosome inactivation [37,38]. These factors are supposed to have different binding patterns in the X chromosome than in autosomes. We therefore analyzed their correlation patterns in X genes and autosomal genes separately. As expected, we found that MES-4 and MRG-4 associate predominantly with autosomal DNAs, while the dosage compensation complex (DCC) subunits bind specifically with X-chromosomal DNAs (data not shown), which is in line with previous reports [19]. Consistent with this finding, MES-4 and MRG-4 show stronger positive correlation with autosomal gene expression.

### Unsupervised clustering reveals general activating and repressing chromatin features for individual genes

As some chromatin features are positively correlated with gene expression levels and some are negatively correlated, the two groups potentially represent general active and repressive marks of gene expression. Yet since these correlations capture only the average behavior across all genes, it is still not clear if these features are strong indicators of the expression levels of individual genes.

**Figure 3:**
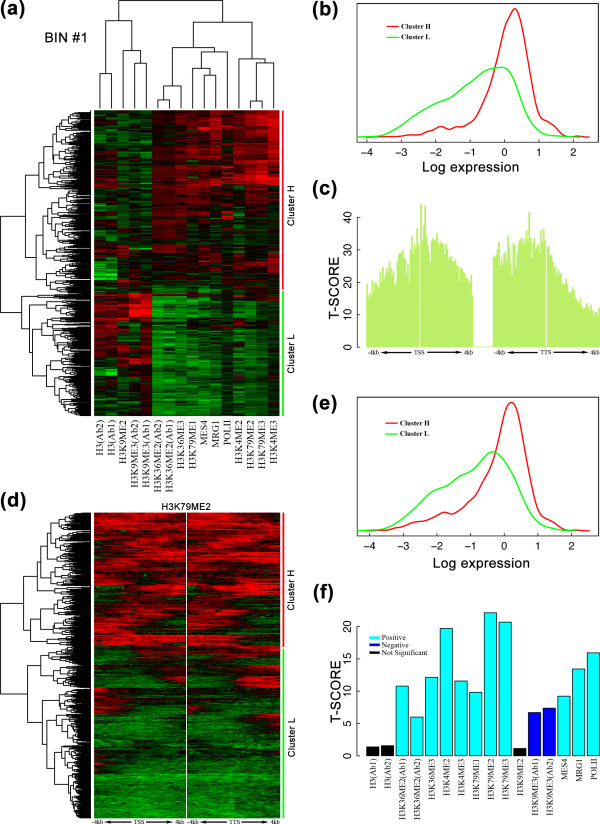
**Hierarchical clustering using either chromatin feature profiles (a-c) or bin profiles (d-f) discriminates highly and lowly expressed genes**. **(a) **Hierarchical clustering of 16 chromatin features in bin 1 (0 to 100 nucleotides upstream of a TSS). The resulting tree is split at the top branch, which divides genes into two clusters, cluster H and cluster L, as labeled. **(b) **Distributions of expression levels of genes in cluster H (red) and cluster L (green). Expression levels are significantly different between the two clusters according to *t*-test (*P *= 3E-202). Expression levels were measured by RNA-seq (see Materials and methods). **(c) **T-scores for the differential expression of the top two gene clusters based on hierarchical clustering of chromatin features in each of the 160 bins. For each bin, hierarchical clustering was performed to separate genes into two clusters. Expression levels between the two clusters were compared and a t-score calculated to measure the capability of the bin to discriminate between genes with high and low expression levels. **(d) **Hierarchical clustering of the genes based on the signal profiles of H3K79me2 across the 160 bins. The resulting tree is also split at the top branch, leading to two gene clusters. **(e) **Distributions of expression levels of genes in the two clusters in (d). The expression levels are significantly different according to *t*-test (*P *= 4E-93). **(f) **T-scores for the differential expression of the two gene clusters based on hierarchical clustering of bin profiles for each individual chromatin feature. Cyan and blue colors indicate a significant positive and negative correlation between a chromatin feature and gene expression levels, respectively. Black color indicates that a chromatin feature could not significantly discriminate between genes with high and low expression levels. To visualize the clustering, 2,000 randomly selected genes are shown. The data for gene expression levels and chromatin features are from the EEMB stage.

In order to examine the relationship between chromatin features and the expression levels of all individual genes, we performed a two-way hierarchical clustering of both the chromatin features and the annotated genes, according to the feature signals at the TSS bins (bin 1). As shown in Figure 3a, genes can be divided into two clusters (labeled as H and L, respectively) based on the signals of the 16 features. We found that the two clusters roughly correspond to genes with high expression levels (H) and genes with low expression levels (L), respectively (Figure 3b). These two clusters are characterized by complementary patterns of chromatin features. Cluster H is characterized by high signals of 11 features (the right component of the upper dendrogram), and low signals for the other 5 features. We note in particular that highly expressed genes tend to have a strong H3K36me3 signal, which is consistent with the role of H3K36me3 as a chromatin mark that activates transcription of associated genes. Similarly, the well-known repressive mark H3K9me3 shows a low signal. Compared to cluster H, genes in cluster L show the opposite pattern of chromatin signals.

To explore which regions around the TSS and TTS provide the greatest power in determining gene expression levels, we repeated the two-way clustering procedure for each of the 160 bins around TSSs and TTSs. Figure 3c shows the resulting t-statistics. We observe that the signals slightly downstream of TSSs are the most informative. In general, the t-statistics decrease as the distance from the TSS or TTS increases. The decay is steeper at the region downstream of TTSs.

The above integrative analysis involves all chromatin features. To examine how each feature individually affects gene expression, for each feature we performed hierarchical clustering of the genes based on the collective signals of the feature at all 160 bins. An example is shown in Figure 3d, in which signals of the single feature H3K79me2 at the different bins were used to cluster the genes. As in the case when all chromatin features were used, the signals from single chromatin features can divide genes into two clusters (that are not exactly the same as, but similar to, the ones obtained from all features) with a significant difference in expression level (Figure 3e). Again we quantified the power of each feature in distinguishing genes with high and low expression levels using t-statistics. As shown in Figure 3f, apart from a few exceptions (black bars), most features are informative. The most informative features are H3K79me2, H3K79me3 and H3K4me2. The informative features can be further grouped into two classes. Activating features are those that are positively correlated with gene expression (cyan) and repressive features are those that are negatively correlated (blue).

### Chromatin features can statistically predict gene expression levels with high accuracy using supervised integrative models

**Figure 4:**
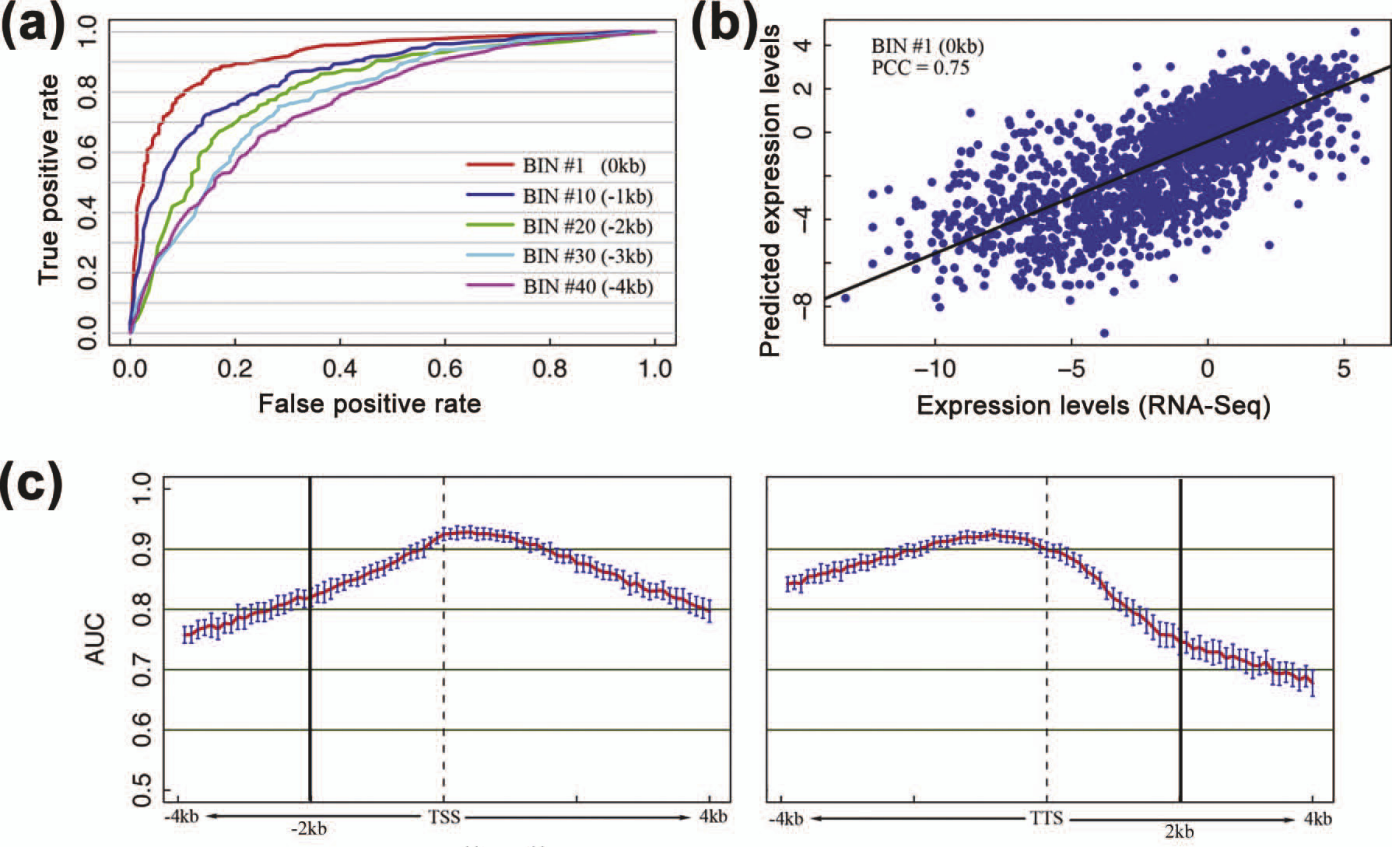
**Prediction power of the supervised models**. **(a) **ROC curves for five different bins based on the results of the SVM classification models. **(b) **Predicted versus experimentally measured expression levels. The SVR regression model was applied to bin 1 for predicting gene expression levels. (PCC, Pearson correlation coefficient). **(c) **The prediction accuracy of SVM classification models for all the 160 bins. For each bin, we constructed an SVM classification model and summarized its accuracy using the AUC score. The AUC scores were calculated based on cross-validation repeated 100 times for each bin. The red curve shows the average AUC scores (mean of 100 repeats) of the bins and the blue bars indicate their standard deviations. The positions of the TSS and TTS are marked by dotted lines.

The above analyses suggest that gene expression levels can be at least partially deduced from chromatin features. To examine how much of gene expression is determined by chromatin features, we tried to predict gene expression levels using the features. We started with the simplified task of distinguishing highly expressed and lowly expressed transcripts, where the two classes of transcripts were constructed by discretizing gene expression levels (see Materials and methods). We divided all the transcripts into training and testing sets, and learned a support vector machine (SVM) model from the signals of all 13 chromatin features of the training transcripts at a certain bin (Figure 1). The model was then used to predict to which class each transcript in the testing set belongs. We repeated the procedure for all 160 bins, and 100 different random splitting of the transcripts into training and testing sets for each bin (see Materials and methods). We represented the overall performance of the model using the receiver operating characteristic (ROC) curve and further quantified the accuracy using the area under the curve (AUC). Figure 4a shows the ROCs corresponding to the prediction performance of five different bins. Compared to random ordering, which would give a diagonal ROC curve on average with an expected AUC of 0.5, we observed that all five curves are much better than random but with diverse performance, which indicates that all the bins are useful to classify gene expression but they are not equally informative. This result is consistent with what we have observed using the unsupervised method described above (Figure 3f). Instead of using SVM, we also learned support vector regression (SVR) models using similar procedures (see Materials and methods) to predict expression values directly. Figure 4b shows that there is a high positive correlation (0.75) between the predicted levels from an SVR model and the actual expression levels measured by RNA-seq. This analysis suggests that chromatin features explain at least 50% of gene expression variation (see Materials and methods).

We then compared the prediction accuracy of all 160 SVM models learned from the different bins. As shown in Figure 4c, the models learned from regions around the TSS (-300 to 500 bp) and upstream of the TTS (-200 bp to 0 bp) have highest accuracy, with AUC values greater than 0.9. Prediction accuracy decreases gradually as we move away from these regions, which confirms the spatial effects that we observed from the unsupervised analysis (Figure 3c).

We have also tested more comprehensive models that combine the chromatin features in 40 bins around the TSS (-2 kb to 2 kb). These comprehensive models achieve slightly higher prediction accuracy than those based on single bins, yet the enhancement is not dramatic, with an average AUC of 0.94 for the classification model (SVM) and an average correlation coefficient of 0.75 for the regression model (SVR) (Figure 6 in Additional file 6).

**Figure 5:**
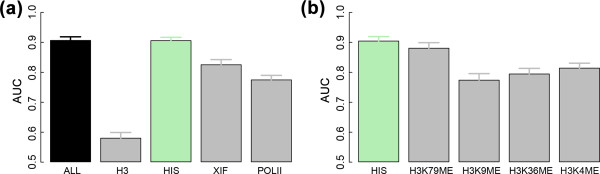
**Prediction power of the SVM models using the signals from different subsets of chromatin features in the 100 nucleotides around the TSS (bin 1). The results are based on cross-validation with 100 trials**. **(a) **ALL, all 21 chromatin features; H3, the two H3 features; HIS, the 11 chromatin modification features; XIF, the seven binding profile features for X-inactivation factors; POLII, the binding profile feature for RNA polymerase II. **(b) **HIS, the 11 chromatin modification features; H3K79ME, H3K79me1, H3K79me2 and H3K79me3; H3K9ME, H3K9me2, H3K9me3(Ab1) and H3K9me3(Ab2); H3K36ME, H3K36me2(Ab1), H3K36me2(Ab2) and H3K36me3; H3K4ME, H3K4me3 and H3K4me3.

We then learned SVM models using only features of individual types. As shown in Figure 5a, the AUC obtained by using all features (black) is comparable to the AUCs obtained from models using only particular subsets of features. Strikingly, the model involving only the 9 histone modification features is almost as accurate as the model involving all 16 features. We further divided the histone modification features into four subsets: modifications on K4, K9, K36 and K79, respectively. While the integrated model with all histone modifications achieves an AUC value of 0.9, using just one of the subsets can yield an AUC higher than 0.8 (Figure 5b). In particular, the set H3K79 is found to be most predictive, which again confirms our previous finding of the importance of these histone modifications in regulating gene expression (Figure 3f).

**Figure 6:**
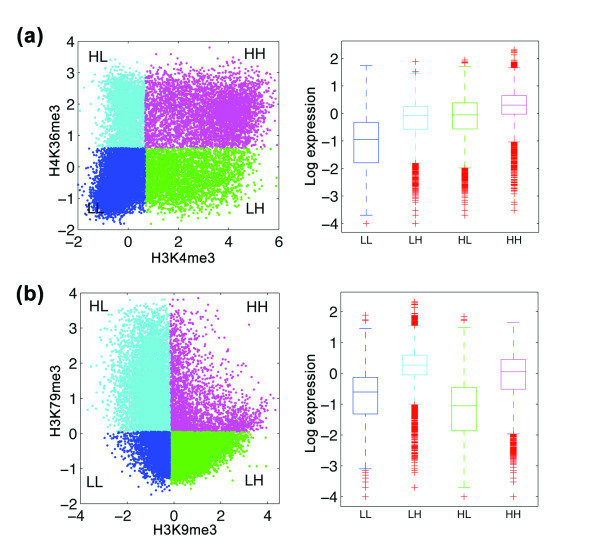
**Co-regulation of transcription by pairs of histone modifications**. **(a) **Categorization of genes into four groups based on signals of H3K4me3 and H3K36me3: HH (magenta), HL (green), LH (cyan) and LL (blue). The signals of histone marks H3K36me3 and H3K4me3 exhibit a bimodal feature. Signals are thus classified into H and L by a Gaussian mixture model. The distributions of expression levels of the four gene groups are shown on the right. **(b) **Same as (a), based on signals of H3K9me3 and H3K79me3. Same as above, the signal of H3K79me3 is again classified by a Gaussian mixture model. The signals of H3K9me3 do not display a bimodal feature; signals are classified into H and L based on whether the value is higher than or lower than the median.

The results of the supervised analysis suggest that chromatin features are not only correlated with expression but are also predictive of the expression levels of individual genes with good accuracy and could explain a large portion of the expression differences between different genes. We note that histone modifications may have other regions of enrichment that are informative about gene expression: for instance, the percentage of gene length with strong histone modification signals. We therefore examined the power of using these features for predicting gene expression levels. Specifically, we calculated the percentage of transcribed regions with strong signals (>10%) for all genes. Using them as predictors, we obtained high prediction accuracy (AUC = 0.90). However, a combination of these percentage features with the original chromatin features does not lead to obvious improvement in prediction accuracy, indicating that they are redundant.

### Combination of chromatin features contribute to gene expression prediction

Both the unsupervised and supervised analyses above suggest that chromatin features possess a certain level of redundancy. In the unsupervised clustering (Figure 3a), different chromatin features show similar signal patterns around the TSS regions of genes. In the supervised predictions (Figure 5), high accuracy was achieved by multiple features as well as feature subsets. Though the SVR model offers good prediction power, it may be instructive to build a simpler linear regression model to explore to what extent the chromatin features are redundant, and to what extent they are interacting in a combinatorial fashion. Specifically, for each bin, we modeled the expression level *y *as a linear combination of the effects of individual histone modification features *x*_*i *_and their products *x*_*i*_*x*_*j*_:

$$y\sim \sum x_{i}+\sum_{i<j}x_{i}x_{j}$$

We found that among the 66 (12 × 11/2) possible interactions between the 12 distinct histone modification features, many interactions are statistically significant. For example, for bin 1, we detected 12 significant interactions (*P *< 0.001, linear regression) between the histone modifications (Table S7 in Additional file 7).

To quantify the importance of these interactions in determining gene expression levels, we compared the above regression model with a singleton model that does not contain the interaction terms:

$$y\sim \sum x_{i}$$

By evaluating the prediction power of the two models using a cross-validation method, we found that with respect to the singleton model the interaction model improves prediction accuracy by 4%. Thus, the contribution of interactions among chromatin features to gene expression prediction is not substantial.

We further examined each pair of modifications individually to see if there is any redundancy between any of the modifications. Using simplified models each involving only two modification features, we found that no two histone modifications are completely redundant (Table S8 in Additional file 8). These results were confirmed by a similar analysis based on mutual information (Figure S9 in Additional file 9). Two examples are shown in Figure 6. In each example, we considered a specific pair of histone modification features, and divided all genes into four categories based on the signals of the two features at their TSS bins. In the first example (Figure 6a), expression levels are the lowest when both H3K4me3 and H3K36me3 are low but moderate if either one of them is high. This suggests that both features are activators. When both features have high signals, an even higher expression level is observed, showing that the two are not totally redundant. In the second example (Figure 6b), H3K9me3 is found to repress gene expression in general, while H3K79me3 is found to activate gene expression. As expected, a combination of high H3K9me3 signal and low H3K79me3 signal results in a lower expression level than when both signals are low. When the signals of both features are high, we observe a significant difference in gene expression compared to the other three cases, indicating that the features contribute to gene expression regulation in a collective manner.

Our analyses of the interactions between the above chromatin features only considered binary interactions between two features. For higher-order relationships involving more features, it is infeasible to perform the same type of analyses, as the number of feature combinations would become intractable. Also, the above analyses only suggest which features interact with each other, but do not explain how the features interact. In particular, the complex correlations between features and gene expression make it difficult to extract directional relationships between them (Figure S10 in Additional file 10). We therefore used Bayesian networks to study the higher order relationships between the chromatin features and gene expression (see Additional file 11 for details).

### The chromatin model is developmental stage-specific

**Figure 7:**
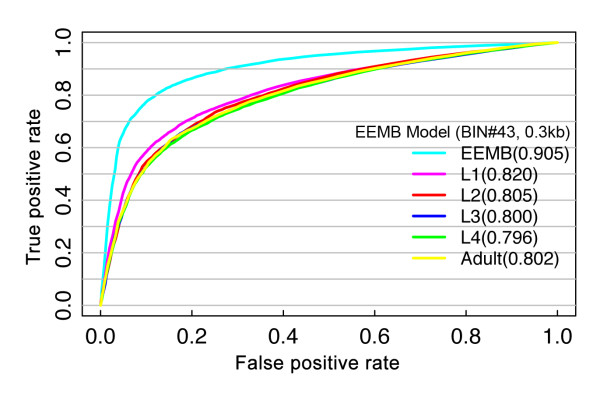
**Developmental stage specificity of the chromatin model**. The EEMB model was constructed using the chromatin features and gene expression data both at the EEMB stage. The model was then used to predict gene expression levels at the EEMB stage and five other developmental stages: L1, L2, L3, L4 and adult. ROC curves are plotted based on the results of 100 trials of cross-validation. For each trial, the dataset was randomly separated into two halves: one half as training data and the other as testing data to estimate the accuracy of the model. The values in parentheses are AUC scores.

We have previously constructed an integrative model using chromatin features at the EEMB stage of *C. elegans *development and used it to predict gene expression levels at the same stage. How well can we predict gene expression levels at other developmental stages using the chromatin feature data from EEMB? To answer this question, we applied the model to predict gene expression at EEMB, L1 (larva stage 1), L2, L3, L4, and adult. Specifically, the chromatin feature data from EEMB were combined with expression data from a stage to train a SVM model, which was then used to predict gene expression levels of other genes at that stage. As shown in Figure 7, the chromatin model based on EEMB data is able to predict the expression at other developmental stages with reasonable accuracy (AUC = 0.8). However, the predictions of gene expression levels in all these stages have lower accuracy than the predictions for EEMB itself. This result suggests that signals from chromatin features are developmental stage-specific and regulate biological processes in a dynamic manner depending on the particular stage. The stage specificity is more apparent when we apply the model to genes that are differentially expressed between stages. For example, we have identified 4,042 genes that differ in expression levels by at least four-fold between EEMB and L3 stages. Using the EEMB stage chromatin model to predict the expression level of these genes, the prediction accuracy further decreases (AUC = 0.70).

### Chromatin features show different correlation patterns with different genes in an operon

**Figure 8:**
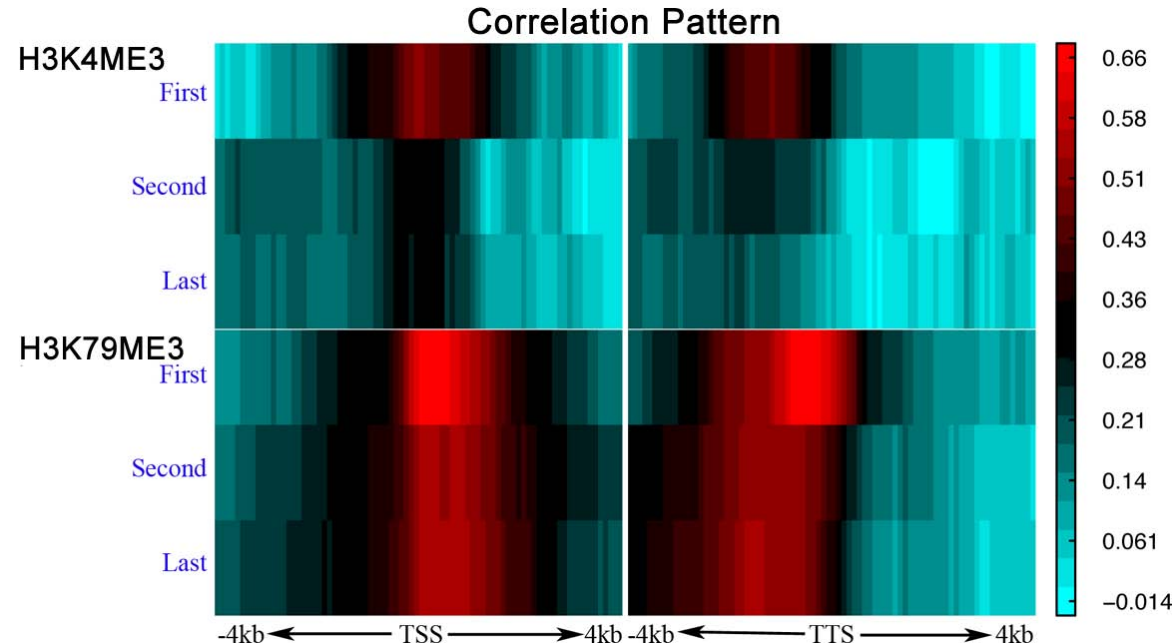
**Correlation patterns of H3K4me3 and H3K79me3 in the 160 bins around the TSS and TTS (from 4 kb upstream to 4 kb downstream) with the expression levels of the first, second and last genes of 881 *C. elegans *operons**.

In *C. elegans *some neighboring genes are organized into operons. The genes in an operon are co-transcribed as a polycistronic pre-messenger RNA and processed into monocistronic mRNAs [39,40]. Here we investigate the differential signals of chromatin features among genes in operons and how this organization affects their expression levels. We collected the first, second and last genes in 881 *C. elegans *operons and calculated the signals of chromatin features in each of the 160 bins around their annotated TSS and TTS. We observed strong correlations between expression levels and chromatin feature signals for the first genes (Figure 8). In comparison, the correlation patterns for the second and last genes of the operons are not as apparent (Figure S12 in Additional file 12). The weaker correlations could be caused by the lack of signals for some histone modification types. As we observed, the mark for active promoters, H3K4me3, demonstrates strong signals around the TSS of the first genes, which is the shared promoter of genes in the same operon. In the upstream region of the internal genes, the H3K4me3 signal is often relatively weak. Alternatively, the weak correlation for internal genes may also be explained by the intensive post-transcriptional regulation of these genes, which can not be captured by our chromatin feature based model [41]. In fact there is only weak correlation (Pearson correlation coefficient (PCC) = 0.10) between the expression levels of the first and the second genes. Moreover, on average the first genes are two-fold and three-fold more highly expressed than the second genes and the last genes, respectively. Taken together, although genes in the operons are co-transcribed, they are regulated post-transcriptionally to achieve distinct expression levels [41].

### Chromatin models learned from protein-coding genes are able to predict microRNA expression levels with high accuracy

Do chromatin features influence transcription of microRNAs in the same way as they do with protein-coding genes? As a way to study the similarity of the two mechanisms, we investigated the effectiveness of the chromatin model learned from protein-coding genes in predicting microRNA expression. Since precise TSSs are not available for most worm microRNAs, we calculated the signals of chromatin features in the genomic regions corresponding to pre-microRNAs, and used them as the input features for our chromatin model.

**Figure 9:**
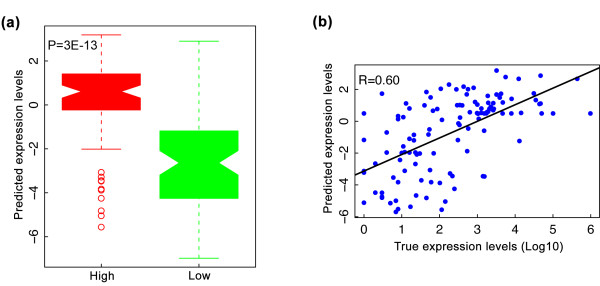
**Prediction of expression levels of microRNAs at the EEMB stage**. **(a) **Predicted expression levels of the experimentally measured highly and lowly expressed microRNAs based on small RNA-seq results. Expression levels of microRNAs at the EEMB stage were predicted using an SVR regression model trained on data for protein-coding genes at the same stage. **(b) **Predicted versus experimentally measured expression levels of microRNAs at the EEMB stage. R is the Pearson correlation coefficient.

We predicted the expression levels of 162 worm microRNAs with genomic locations obtained from miRBASE [42]. We then compared our predictions with the experimental measurements performed by Kato *et al. *[43]. As shown in Figure 9, our predictions are in good agreement with the experimental results in the EEMB stage (see also the prediction results for the L3 stage in Figure S13 in Additional file 13). Some microRNAs locate within or near gene loci, which may confound the prediction of microRNA expression. To address this issue, we also checked the prediction accuracy using only microRNAs that are away from any known gene, and obtained similar prediction accuracy (PCC = 0.62).

It is interesting to see that the expression of microRNAs can be accurately predicted using a chromatin model trained by data for protein-coding genes. Consistent with previous reports on microRNA transcriptional regulation [44,45], this result suggests that microRNAs and protein-coding genes share a similar mechanism of transcriptional regulation by chromatin modifications.

As with the prediction of expression levels of protein-coding genes, the prediction accuracy of microRNA expression also shows developmental stage specificity. When the signals of the chromatin features from the EEMB stage were used, the resulting model achieved the best accuracy when predicting microRNA expression at the same stage (PCC = 0.60), whereas for stages L1, L2, L3, L4 and adult, the accuracy is much lower (PCC < 0.50) (Figure S14 in Additional file 14). Similarly, when chromatin features at L3 were used to train the model, the model achieved better prediction results in L3 than in other stages.

### Application to other organisms

**Figure 10:**
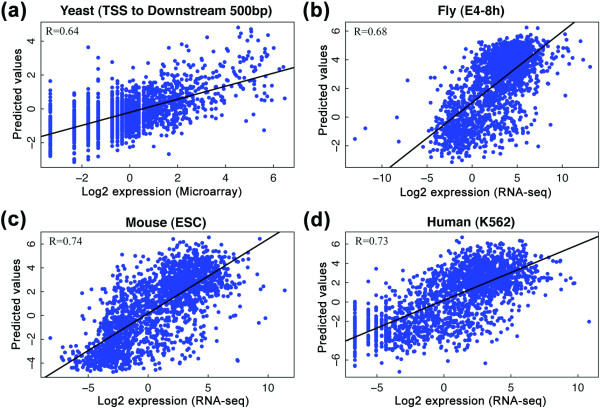
**Prediction accuracy of the chromatin model in four other species**. **(a-d) **Expression levels of genes are predicted using the SVR method. In yeast, average signals of chromatin features from the TSS to 500 bp upstream were used as predictors (a); in the other species, signals of chromatin features within the bin at the TSS (bin 1) were used as predictors (b-d). E4-8 h: embryonic stage at 4 to 8 h; ESC, embryonic stem cell.

The models described above provide a useful tool to integrate gene expression and chromatin data. Currently, the *C. elegans *dataset is the best one to demonstrate the utility of the method and we have focused on it here. However, we know that further integrated genomic datasets (comprising matched genome-wide histone features and expression measurements) are coming in many other organisms. Thus, to illustrate the broad utility of our method, we demonstrate here how readily it can be applied in other contexts. Specifically, we have packaged our methods as a tool and applied it to data sets from four other organisms: yeast, fruit fly, mouse and human. The results indicate that chromatin features, in particular histone modifications, are highly correlated to gene expression levels in all these organisms (Figure 10). More importantly, the relative statistical contribution of each histone modification type to expression is similar in tested organisms (and also in different tissues, cell-lines, and developmental stages). For example, H3K4me3 signals around the TSS of genes show high predictive capability in all the analyses we have performed. We also found that the models based on expression levels measured by RNA-seq achieved higher prediction accuracy than those by microarrays, consistent with the higher measurement accuracy of RNA-seq compared to microarrays. Our method can, of course, be applied to multiple data sets in each species (for example, different developmental stages in fruit fly). Figure 10 shows only a single illustrative example for each species. We only show initial statistical analysis here, further biological interpretation would, of course, be the subject of future studies.

## Discussion

In this study, we present a systematic analysis of the genome-wide relationship between chromatin features and gene expression. We have shown that, in terms of gene expression prediction, information from different histone modification features is considerably redundant. Here in this paper, we use the modENCODE worm data to exemplify our analysis. In fact, we have applied our methods to two other histone modification data sets: human CD4+ T-cell data [46] and mouse embryonic stem cell deta [47]. In both data sets, we found that histone modifications account for more than 50% of variation of gene expression and distinct modification types were redundant for predicting gene expression levels. This is consistent with a recent study by Karlic *et al. *[48] performed in human CD4+ T cells.

The existence of a 'histone code' has been intensively debated since the time that the hypothesis was first proposed 10 years ago [24,25]. Previous studies have demonstrated both pros and cons for the hypothesis [11,28,49,50]. Indeed, for some specific genes, it has been demonstrated that the patterns of a subset of histone marks could be viewed as an accurate predictor of gene regulation in non-trivial manners [50]. Nevertheless, the readout of these patterns is largely gene specific and dependent on the cellular context, which makes it difficult for these cooperative effects to be viewed as a universal 'code'. Therefore, by using the term histone code, we might have underestimated the complexity and over-generalized the meaning of chromatin modifications and their roles in biological processes. On the other hand, at a global level, previous studies have reported substantial correlations among distinct chromatin features [13,14,17,28,51]. These results, and the information redundancy we observed, are consistent with the simple 'histone code' argument [28], in which the combinatorial effects are cumulative rather than synergistic.

We have shown that chromatin features are strongly correlated with gene expression. Nevertheless, it should be noted that our models could not reveal if histone modifications are the 'cause' or 'consequence' of transcription. In fact, both directions of causality have been previously reported. Some studies have proposed that some histone modifications are the memory of past transcriptional events resulting from previous active transcription [52-54]. For instance, it has been shown that phosphorylation in the tail of Pol II is required for H3K4me3, revealing that it is a direct consequence of Pol II passing through the TSS [55]. Other studies, however, have shown that chromatin modification changes precede changes in gene expression [56]. A recent study in human T cells suggested that, for both protein-coding and miRNA genes, activating histone marks were already in place before induction of expression, and these marks were maintained even after the genes were silenced [45]. This finding shows that histone modification can be both cause and consequence of gene transcription, and that a full explanation will require incorporation of additional data. Generalizing our model to follow a time course of changing histone modifications might be helpful for understanding this issue.

The supervised chromatin model trained from expression data for protein-coding genes can accurately predict the abundance of both protein-coding genes and microRNAs, which suggests that microRNAs and protein-coding genes share similar mechanisms of transcriptional regulation by chromatin modifications [44,45]. To predict the expression levels of microRNAs, we used the signal of chromatin features around the start sites associated with pre-microRNAs, which might be several kilobases from the actual TSS of microRNA genes. Despite this caveat, our model still achieved high prediction power. We expect to obtain more accurate predictions if more precise annotation for microRNA genes becomes available in the future.

In summary, we have presented a series of supervised and unsupervised methods for analyzing multiple aspects of the regulation of gene expression by chromatin features. Apart from predicting gene expression, these methods can be used to address important biological questions such as combinatorial regulation and microRNA transcription. These and other statistical methods will be essential to gaining new understanding of biological processes from the tremendous amount of data that will soon be made available by large collaborative projects such as modENCODE.

## Materials and methods

### Datasets and gene annotation

Expression levels for all annotated worm transcripts at different stages of development, including EEMB, mid-L1, mid-L2, mid-L3, mid-L4 and young adult stages, were quantified using RNA-seq. Pol II binding across the genome at different stages was profiled using ChIP-seq. All the other chromatin features were profiled using ChIP-chip experiments. These chromatin features include histone H3 occupation, histone methylations (H3K4me2, H3k4me3, H3K9me2, H3k9me3, H3k27me3, H3K36me2, H3K36me3, H3K79me1, H3K79me2 and H3K79me3), binding of dosage compensation complex (DCC) proteins (SDC2, SDC3, DPY27, DPY28 and MIX1) and other X-chromosome inactivation factors (MES4 and MRG1). For some chromatin features such as H3K9me3, biological replicates using different antibodies were available. Profiles of these chromatin features were measured for different developmental stages, in particular at EEMB and L3 stages. A list of the data, with their Gene Expression Omnibus IDs can be found in Additional file 15. All these data are available from the modENCODE website at [57]. Operon information for *C. elegans *was obtained from a previous study by Blumenthal *et al. *[39]. The dataset contains a total of 881 operons with 2.6 genes in each of them on average.

MicroRNA expression levels at different developmental stages of *C. elegans *were obtained from small RNA-seq measurements performed by Kato *et al. *[43]. Annotation of worm transcripts was downloaded from WormBase at [58,59]. Annotation of nematode microRNAs was downloaded from the microRNA database miRBASE at [42,60]. Assembly version WS180 of *C. elegans *was used for gene and microRNA annotations and data processing of all the chromatin features.

### Binning DNA regions

We obtained the genomic locations and structures of 27,310 protein-coding transcripts of *C. elegans *from WormBase. The contribution of each chromatin feature to gene expression is thought to be affected by many factors, in particular its position relative to the TSS. We therefore divided the DNA region from 4 kb upstream to 4 kb downstream of the TSS of each transcript into 80 small bins, each of 100 bp in size. The DNA region around the TTS of each transcript was also divided into 80 100-bp bins. For each bin, we calculated the average signal of each chromatin feature across all transcripts. Specifically, for chromatin features profiled by ChIP-chip experiments, the signals of the probes that fall into a bin were averaged. For features profiled by ChIP-seq experiments, the number of reads that cover a bin was counted and weighted according to their overlap with the bin. We note that for short transcripts less than 8 kb in length, some bins around the TSS and TTS overlap, and for transcripts representing alternative splicing isoforms of the same gene or located close to each other in the genome, their bins can also overlap. To ensure these issues do not affect our main findings, we have performed analysis using only genes that are longer than 8 kb and genes that are far away from coding genes (see main text). It should also be noted that the precise TSS and TTS of worm transcripts are largely unknown and the locations used here usually represent the start and end positions of the protein-coding regions.

### Hierarchical clustering

The data processing described above results in a matrix A_n × m _for each of the 160 bins, where n is the number of transcripts and m is the number of chromatin features. To make the signals for different chromatin features comparable, we normalized the columns of A by subtracting the median and then divided by the standard deviation of each column across all transcripts. We performed hierarchical clustering analysis using the normalized matrix for a given bin. To evaluate the capability of a bin to discriminate between genes with high and low expression levels, we divided the transcripts into two clusters by splitting the resulting hierarchical tree at the top level. The expression levels of transcripts in the two clusters measured by RNA-seq experiments were compared using *t*-test. We repeated this procedure for all 160 bins, which resulted in a t-score for each bin. Those t-scores reflect the capability of chromatin features in these bins to separate genes with low and high expression levels.

Similarly, given a specific feature, we performed hierarchical clustering using its signals across all 160 bins. The clustering analysis was conducted for all chromatin features, and the capability of each feature to predict gene expression was evaluated and compared by their t-scores calculated as described above.

### Supervised models for gene expression prediction

We constructed supervised learning models to integrate the chromatin features for gene expression prediction. In principle, the chromatin features of each of the 160 bins could contribute to regulation of gene expression. We therefore constructed the model in a bin-specific manner to investigate the relative importance of each bin for regulation of gene expression. We devised both classification and regression models, implemented by using the SVM and SVR [61] methods, respectively.

In the classification model the expression levels of transcripts at a particular developmental stage (measured by RNA-seq and quantified as RPKM (reads per kilobases per million mapped reads)) were discretized into two classes, with high and low expression level, respectively, by setting the median expression levels as the cutoff values. The chromatin features in a given bin were then used as classifiers to predict the two classes. The prediction power of the classification model was evaluated using cross-validation. Specifically, we split the whole dataset into two halves, the training data and the testing data. The SVM model was first trained on the training data and then used to predict the classes of expression levels of the transcripts in the testing data. The predicted classes at various thresholds were compared with their actual classes to calculate the sensitivity (also called true positive rate, the proportion of actual positives that are correctly identified) and specificity (also called true negative rate, the proportion of negatives that are correctly identified). The tradeoff between sensitivity and specificity can be best visualized as a graphical plot of the sensitivity against 1 - specificity, which is called a ROC curve. The area under the ROC curve (AUC) is a frequently used summary statistic for measuring the prediction power of classification models.

In the regression model, we directly predicted the expression levels of transcripts rather than classifying them into two broad expression categories. The prediction power of the regression model was also checked using cross-validation. The SVR model was trained on the training data and applied to the testing data. Then the predicted expression levels for transcripts in the testing data were compared with their actual levels measured by RNA-seq experiment. The correlation between predicted and actual expression level indicates the prediction power of the model.

In a linear regression model, the square of the correlation (R^2^) between the predicted values and the actual values is equal to the fraction of total variance in the observed data explained by the predictions. We used this quantity to estimate how much variation of gene expression can be explained by the chromatin features.

To estimate the predictive power of classification and regression models for each of the 160 bins, we repeated the cross-validation procedure 100 times. The mean and standard deviation of the resulting 100 AUC scores were calculated for each bin as a measurement of the predictive power of the SVM classification model. Similarly, the accuracy of the SVR model for a bin was reflected by the mean and standard deviation of the 100 correlation coefficients.

### Detecting combinatorial effects of chromatin features using linear models

To investigate the interaction between chromatin features, we constructed and compared the following two linear models:

$$y\sim \sum x_{i}+\sum_{i<j}x_{i}x_{j}\;\left(\text{Interaction model}\right)$$

$$y\sim \sum x_{i}\;\left(\text{Singleton model}\right)$$

The Interaction model takes into account the interaction terms. Based on the Interaction model, we identified significant interactions in each bin.

The power of the two models for predicting gene expression was evaluated by cross-validation. Data were randomly split into training and testing data sets. The models were trained on the training model and then applied to the testing data for validation. The accuracy of the models was measured by the correlation between predicted expression levels and experimental measurement.

To investigate the interactions among pairs of chromatin features, we constructed the simplified models involving only two features:

$$y\sim x_{i}+x_{j}+x_{i}x_{j}$$

A significant interaction term would indicate that the interaction between the two features has a significant effect on gene expression.

### Predicting expression levels of microRNAs

We downloaded the annotation of 162 *C. elegans *microRNAs from the miRBASE database [42]. For most microRNAs, the annotation provides no information about the TSSs. Instead, only the start and end positions of the corresponding pre-microRNAs (about 100 nucleotides in length) are available. To predict the expression levels of microRNAs, we calculated the signals of all chromatin features within the associated pre-microRNAs and applied our model trained on chromatin features associated with protein-coding genes. We applied both the SVM classification and the SVR regression models to predict microRNA expression. The resulting predictions were validated using measured microRNA expression levels from small RNA sequencing performed by Kato *et al. *[43].

### Data sets for other organisms

In yeast, the expression levels of genes were measured by microarrays and available from Wang *et al. *[62]; the histone modification data are performed by Pokholok *et al. *[63]. In fruit fly, the gene expression and chromatin data at 12 different developmental stages were obtained by using RNA-seq and ChIP-seq experiments, respectively, which are available from the modENCODE website at [57]. In mouse, the expression data for embryonic stem cells and neural progenitor cells were from Cloonan *et al. *[64]; and the histone modification data for matched cell lines were obtained from Mikkelsen *et al. *[47] and Meissner *et al. *[65]. In human, the gene expression data in K562 and GM12878 cell lines were performed by Mortazavi *et al. *[66], and chromatin data were downloaded from the ENCODE project at [2,67].

### Availability of our code

All the analysis described in this paper was performed using the R package. The related R code and example data sets are available for download from [68].

## Abbreviations

AUC: area under the curve; bp: base pairs; ChIP: chromatin immunoprecipitation; ChIP-chip: ChIP-on-chip; ChIP-Seq: ChIP-sequencing; EEMB: early embryonic; modENCODE: model organism encyclopedia of DNA elements; PCC: Pearson correlation coefficient; Pol II: RNA polymerase II; RNA-seq: RNA-sequencing; ROC: receiver operating characteristic; SVM: support vector machine; SVR: support vector regression; TSS: transcription start site; TTS: transcription termination site.

## Authors' contributions

CC and MG conceived and designed the study. CC and KKY performed the full analysis. CC, KKY, KYY, RA, JR, CS and MG wrote the manuscript.

## Supplementary Material

Additional file 1 **Signal patterns of Pol II around TSS and TTS regions (from -4 kb to 4 kb) at different developmental stages**. At each stage, the signals were normalized by subtracting the average and then divided by the standard deviation of the signals over all the 160 bins. The location of the TSS and TTS are marked as dotted lines. Click here for file

Additional file 2 **Correlation patterns of chromatin features with gene expression at the EEMB stage based on long transcript genes only**. Only genes longer than 8 kb were used for correlation computations so that there is no overlap between the TSS and TTS bins. Click here for file

Additional file 3 **Correlation patterns of chromatin features with gene expression at the EEMB stage based on transcripts that are far away from any other transcripts**. Only the transcripts that are at least 4 kb away from any other transcripts were used for correlation computations so that there is no overlap between bins of nearby transcripts. Click here for file

Additional file 4 **Correlation patterns of chromatin features with gene expression at the L3 stage**. Correlation was calculated based on long transcripts (>8 kb). Click here for file

Additional file 5 **Correlation patterns of chromatin features with gene expression at the EEMB stage based on single-transcript genes only**. Click here for file

Additional file 6 **Prediction of gene expression using chromatin features in all the 40 bins around the TSS (from -2 kb to 2 kb)**. **(a) **ROC curve of the SVM classification model. **(b) **Predicted expression levels versus actual expression levels measured by RNA-seq experiment. PCC, Pearson correlation coefficient. Click here for file

Additional file 7 **Interaction between all possible pairs of histone modifications**. Interaction between all possible pairs of histone modification as indicated by linear model in bin 1. For each pair, both the results of linear models with the interaction terms (Interaction models) and without the interaction terms (Singleton models) are shown. Click here for file

Additional file 8 **The significant interactions between chromatin features based on a linear model**. The significant interactions between chromatin features based on a linear model with 12 different chromatin features and their pairwise interaction terms. Click here for file

Additional file 9 **Mutual information between expression and pairwise histone modification signals**. For each pair of histone modifications (denoted as H1, H2), the heat map shows the normalized mutual information I(E, H1 AND H2)/max(I(E,H1),I(E,H2)). For pairs such as H3K4me2 and K4K36me3, the combination of two features gives a higher predictive power than the two individual features. Click here for file

Additional file 10 **Interactions among chromatin features and expression**. **(a) **Node colors indicate the correlation of the corresponding features with gene expression. Edge colors indicate the correlation between the two connected features. Only interactions with a strong correlation (|PCC| >0.3) are shown. **(b) **The directional relationships inferred from Bayesian network analysis. Arrow sizes indicate the confidence scores of the directed edges. Only interactions with a confidence score (combined for both directions) of at least 80% are shown. Click here for file

Additional file 11 **Supplementary documents about the Bayesian network analysis and so on**. The file contains additional information about the Bayesian network analysis. Click here for file

Additional file 12 **Correlation patterns of chromatin features in 40 bins around the TSS and TTS (from -2 kb to 2 kb) of the first and the second genes in 881 worm operons**. Click here for file

Additional file 13 **Predicted expression levels of microRNAs at stage L3**. MicroRNAs are divided into high (red) and low (green) groups based on their measured expression levels in small RNA-seq experiments. Click here for file

Additional file 14 **Stage specificity of chromatin models for microRNA expression predictions**. The chromatin model was trained using the chromatin and expression data of protein-coding genes at the EEMB stage. The model was then used to predict microRNA expression levels at six stages. R indicates the Pearson correlation coefficient between the predicted expression levels and the actual expression levels from RNA-seq experiments. Click here for file

Additional file 15 **Gene Expression Omnibus accession ID of data sets used in this work**. Click here for file
