# Supplementary figures and images for: A statistical framework for modeling gene expression using chromatin features and application to modENCODE datasets
Source: Genome Biol. 2011 Feb 16;12(2):R15. doi: 10.1186/gb-2011-12-2-r15 (PMC3188797; doi:10.1186/gb-2011-12-2-r15)

# Signal Pattern (Pol II)

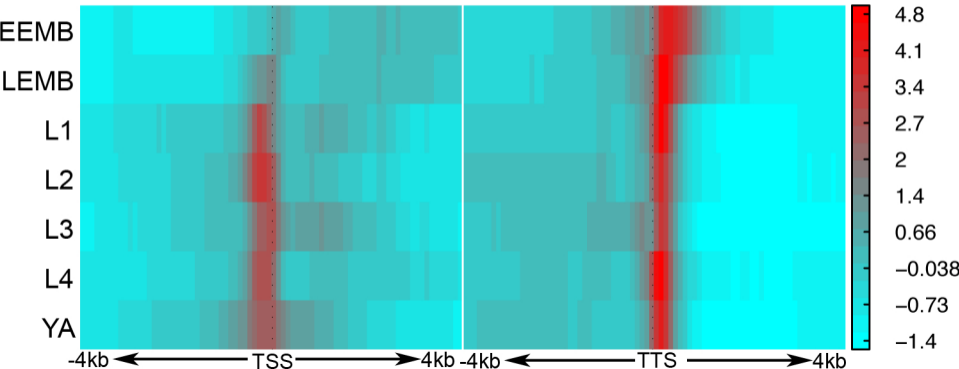

Supplement: Additional file 1 — Signal patterns of Pol II around TSS and TTS regions (from -4 kb to 4 kb) at different developmental stages. At each stage, the signals were normalized by subtracting the average and then divided by the standard deviation of the signals over all the 160 bins. The location of the TSS and TTS are marked as dotted lines. [file gb-2011-12-2-r15-S1.PDF]

Correlation Pattern

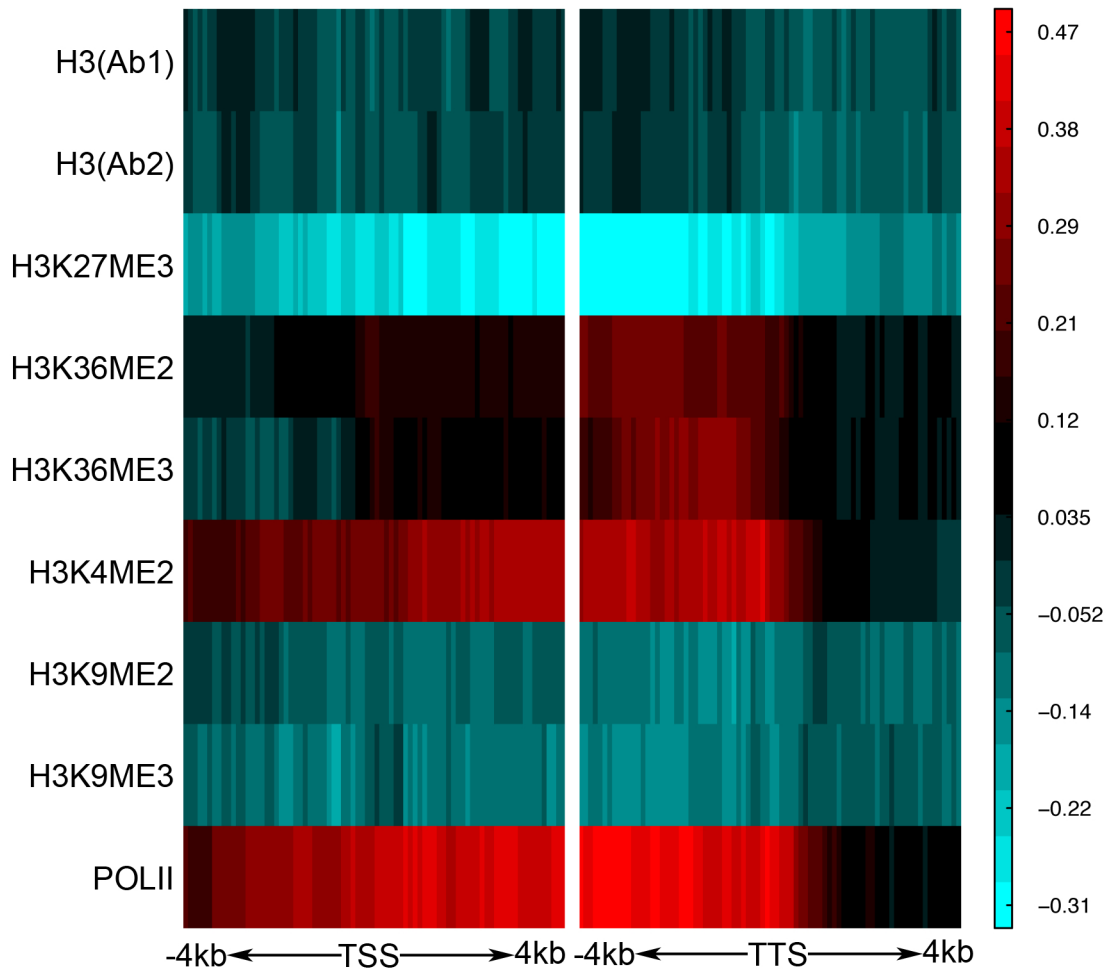

Supplement: Additional file 2 — Correlation patterns of chromatin features with gene expression at the EEMB stage based on long transcript genes only. Only genes longer than 8 kb were used for correlation computations so that there is no overlap between the TSS and TTS bins. [file gb-2011-12-2-r15-S2.PDF]

Correlation Pattern

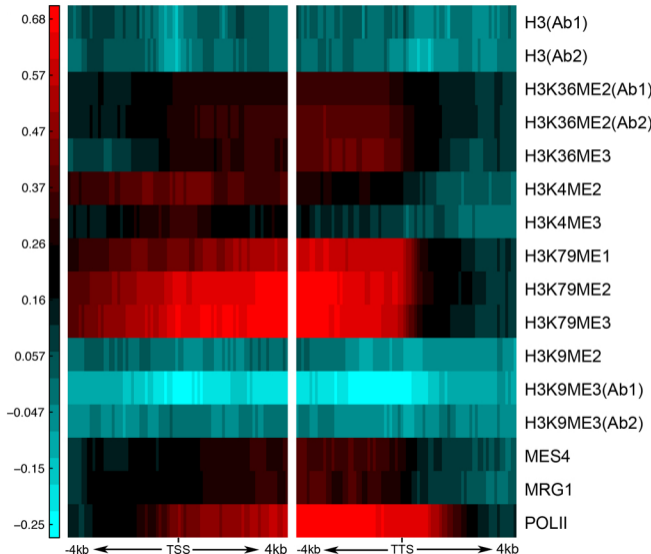

Supplement: Additional file 3 — Correlation patterns of chromatin features with gene expression at the EEMB stage based on transcripts that are far away from any other transcripts. Only the transcripts that are at least 4 kb away from any other transcripts were used for correlation computations so that there is no overlap between bins of nearby transcripts. [file gb-2011-12-2-r15-S3.PDF]

Correlation Pattern

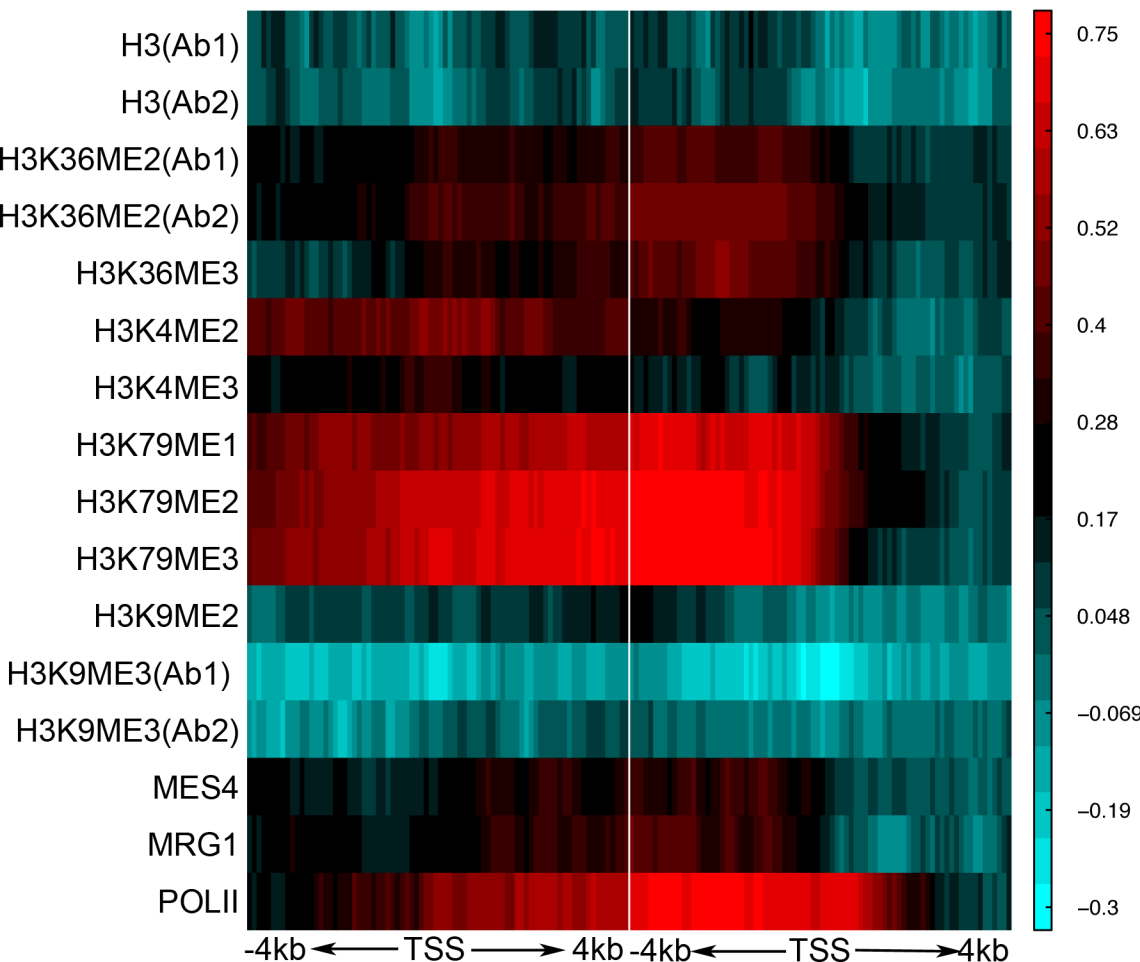

Supplement: Additional file 4 — Correlation patterns of chromatin features with gene expression at the L3 stage. Correlation was calculated based on long transcripts (>8 kb). [file gb-2011-12-2-r15-S4.PDF]

Correlation Pattern

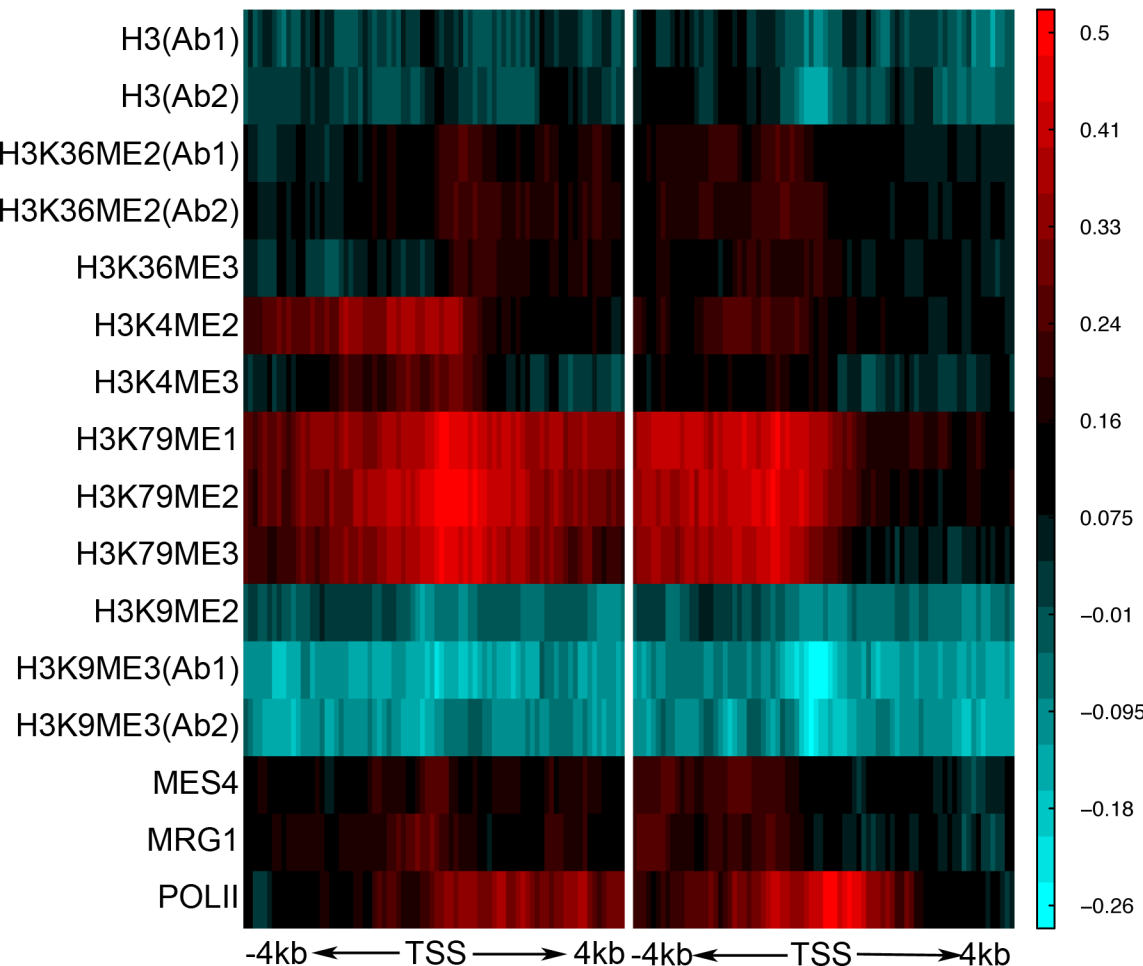

Supplement: Additional file 5 — Correlation patterns of chromatin features with gene expression at the EEMB stage based on single-transcript genes only. [file gb-2011-12-2-r15-S5.PDF]

**A**

Sensitivity

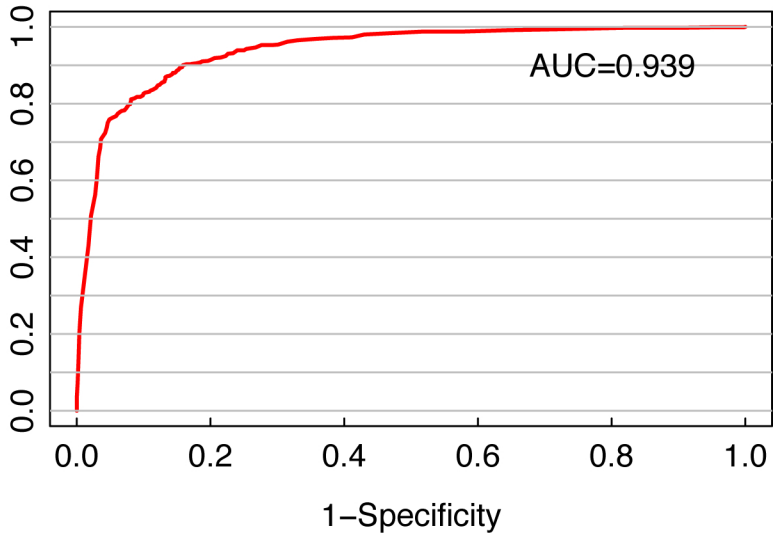**B**

Predicted Expression Levels

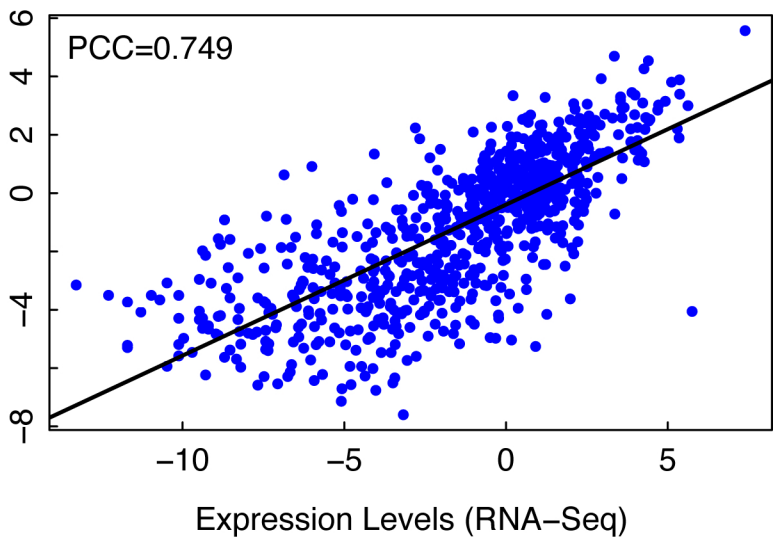

Supplement: Additional file 6 — Prediction of gene expression using chromatin features in all the 40 bins around the TSS (from -2 kb to 2 kb). (a) ROC curve of the SVM classification model. (b) Predicted expression levels versus actual expression levels measured by RNA-seq experiment. PCC, Pearson correlation coefficient. [file gb-2011-12-2-r15-S6.PDF]

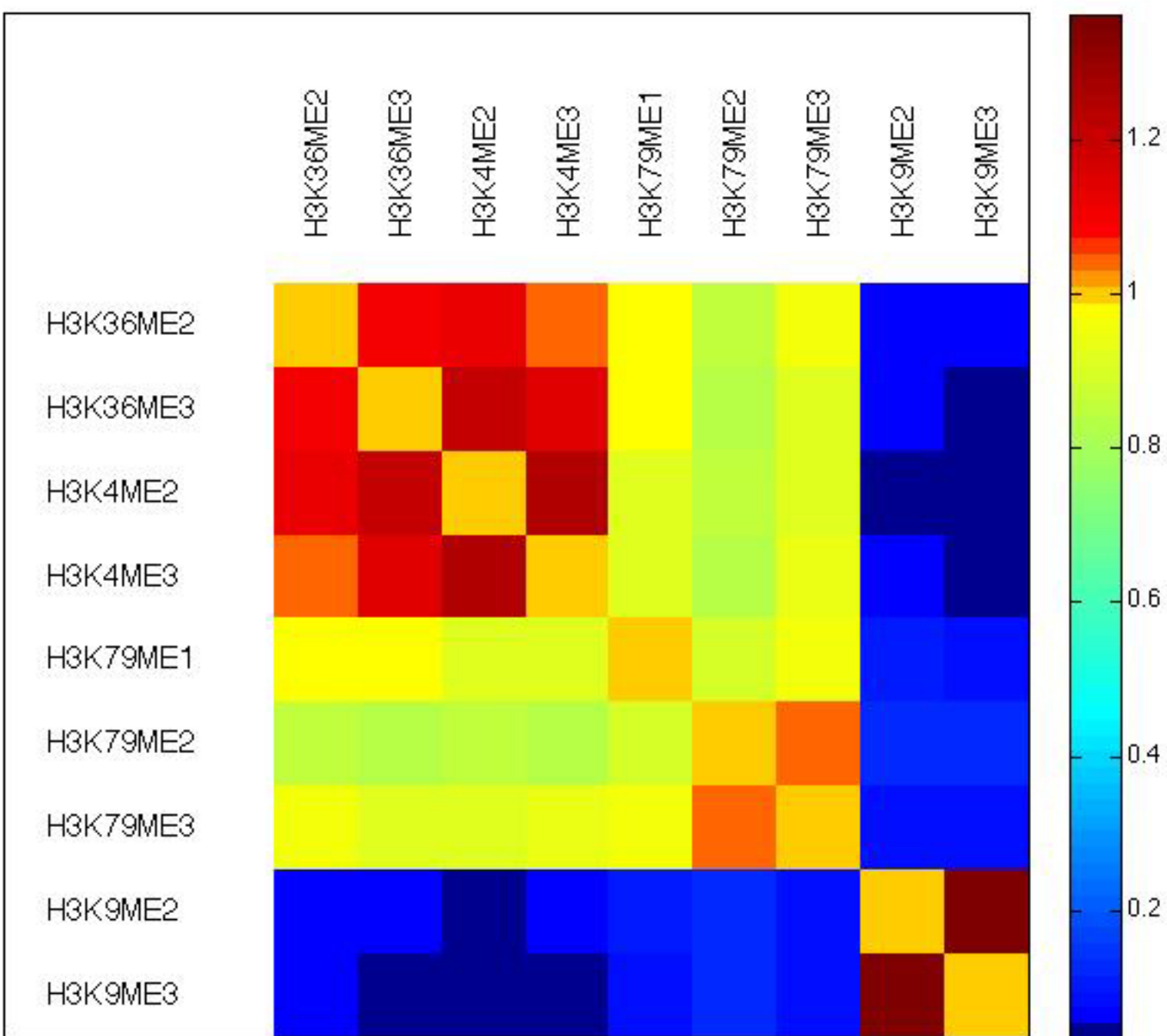

Supplement: Additional file 9 — Mutual information between expression and pairwise histone modification signals. For each pair of histone modifications (denoted as H1, H2), the heat map shows the normalized mutual information I(E, H1 AND H2)/max(I(E,H1),I(E,H2)). For pairs such as H3K4me2 and K4K36me3, the combination of two features gives a higher predictive power than the two individual features. [file gb-2011-12-2-r15-S9.PDF]

**(a)**

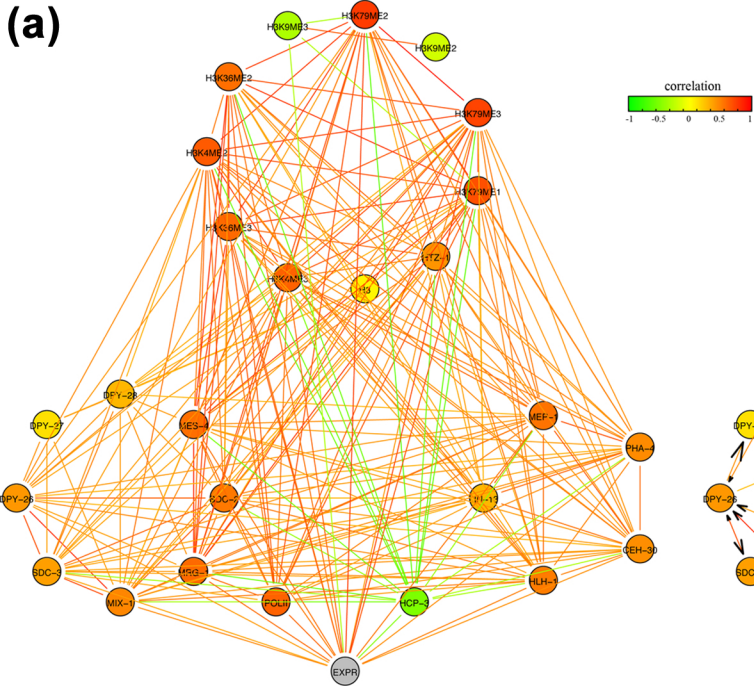

**(b)**

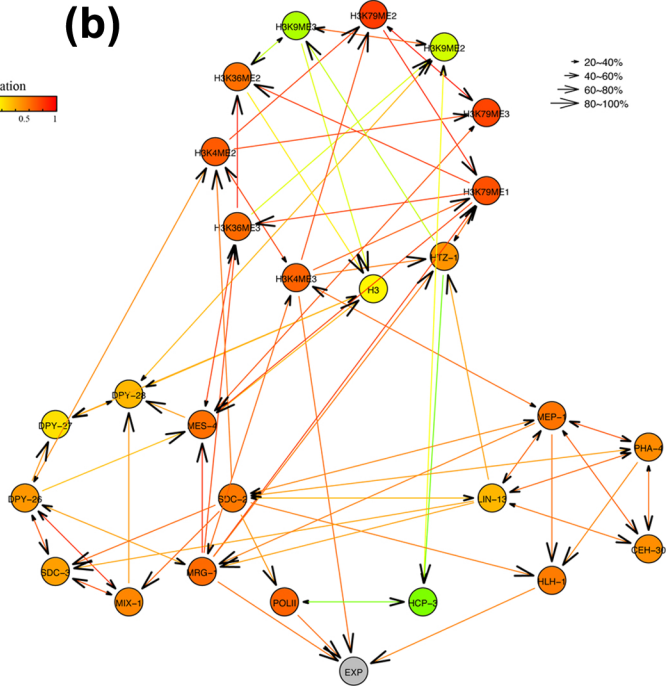

Supplement: Additional file 10 — Interactions among chromatin features and expression. (a) Node colors indicate the correlation of the corresponding features with gene expression. Edge colors indicate the correlation between the two connected features. Only interactions with a strong correlation (|PCC| >0.3) are shown. (b) The directional relationships inferred from Bayesian network analysis. Arrow sizes indicate the confidence scores of the directed edges. Only interactions with a confidence score (combined for both directions) of at least 80% are shown. [file gb-2011-12-2-r15-S10.PDF]

**L3**

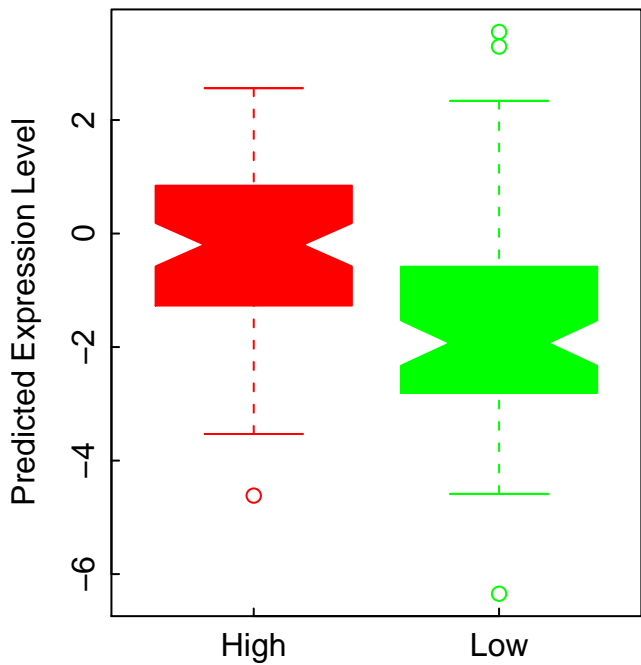

Supplement: Additional file 12 — Correlation patterns of chromatin features in 40 bins around the TSS and TTS (from -2 kb to 2 kb) of the first and the second genes in 881 worm operons. [file gb-2011-12-2-r15-S12.PDF]

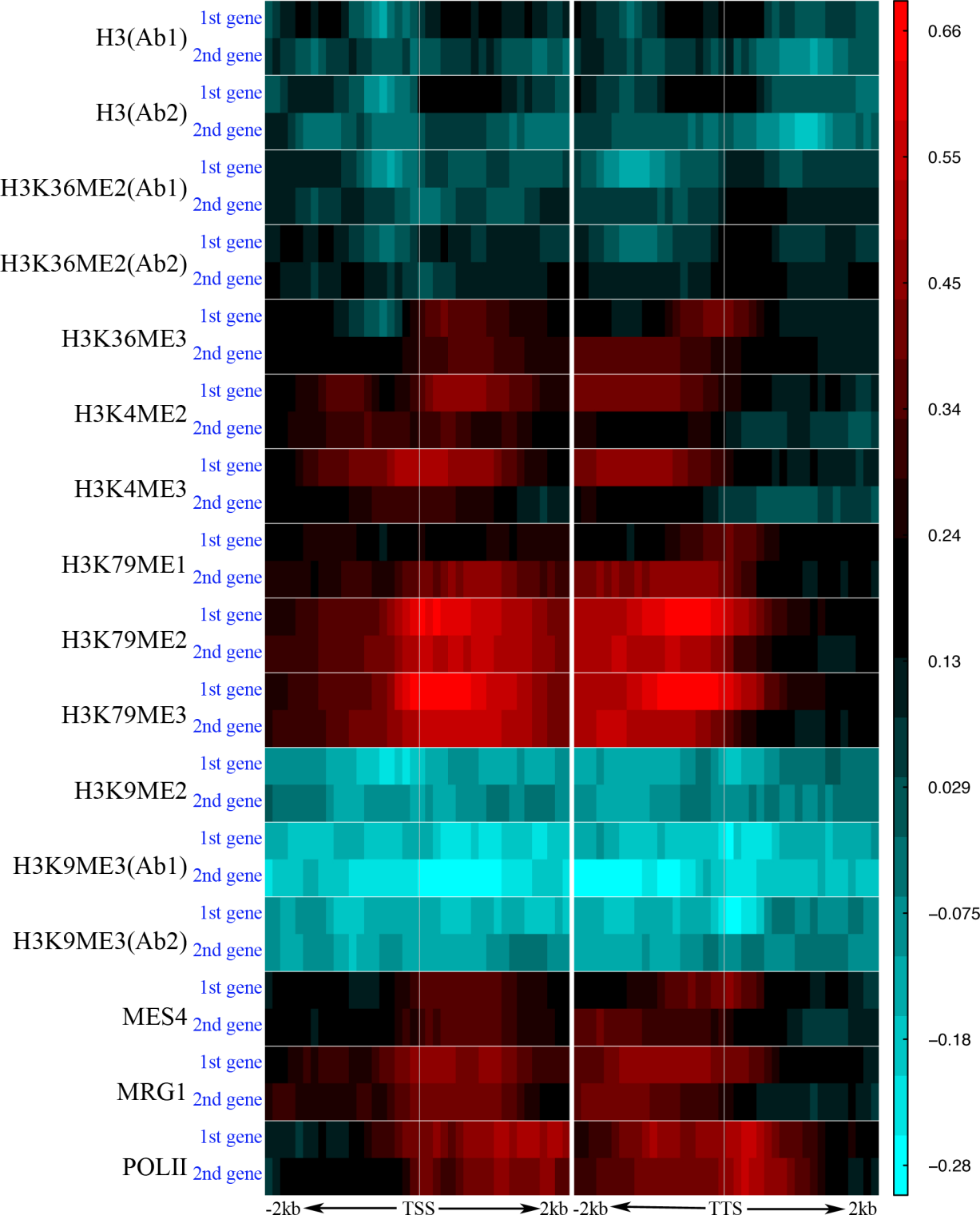

Supplement: Additional file 13 — Predicted expression levels of microRNAs at stage L3. MicroRNAs are divided into high (red) and low (green) groups based on their measured expression levels in small RNA-seq experiments. [file gb-2011-12-2-r15-S13.PDF]

**EEMB (R=0.595)**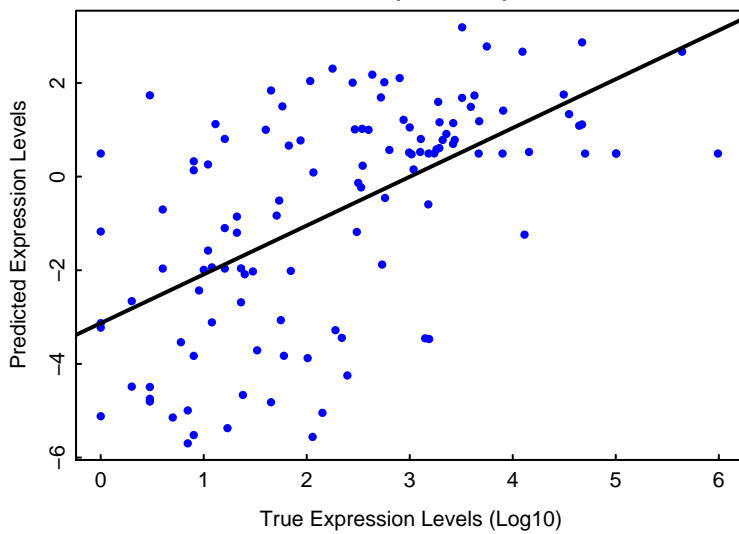**L1 (R=0.427)**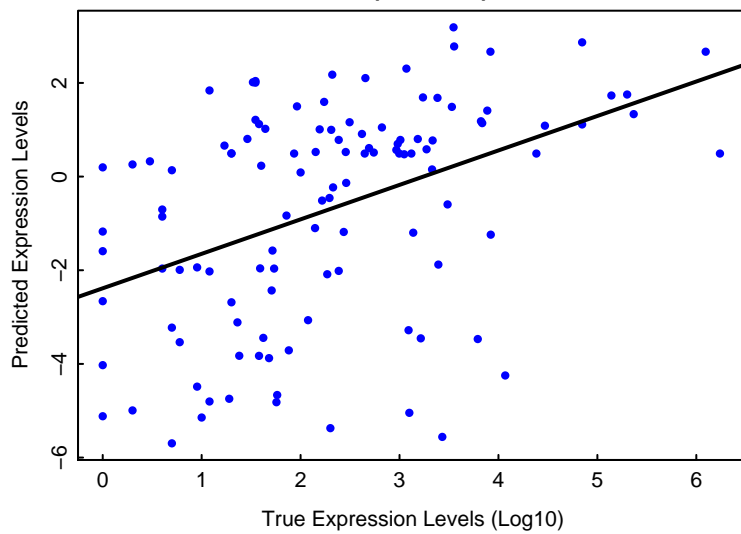**L2 (R=0.428)**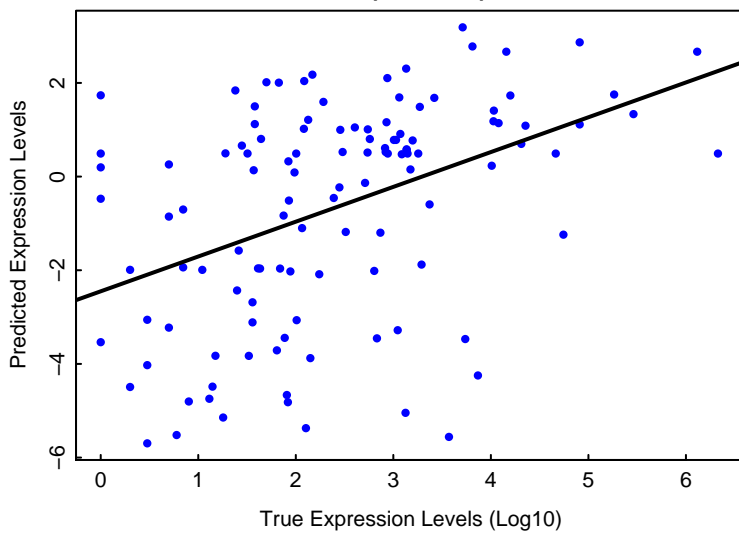**L3 (R=0.428)**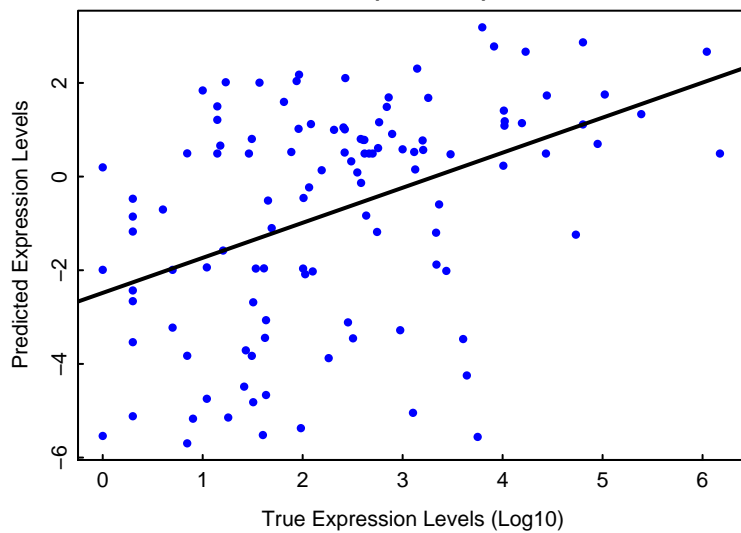**L4 (R=0.483)**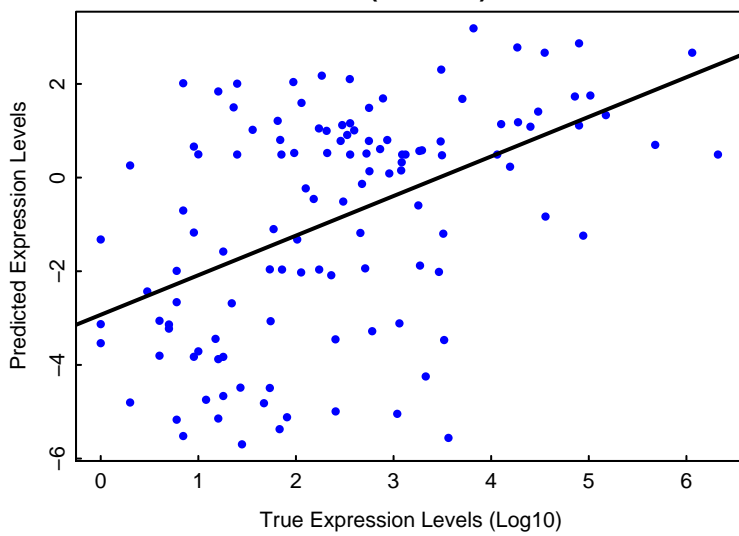**Young Adult (R=0.482)**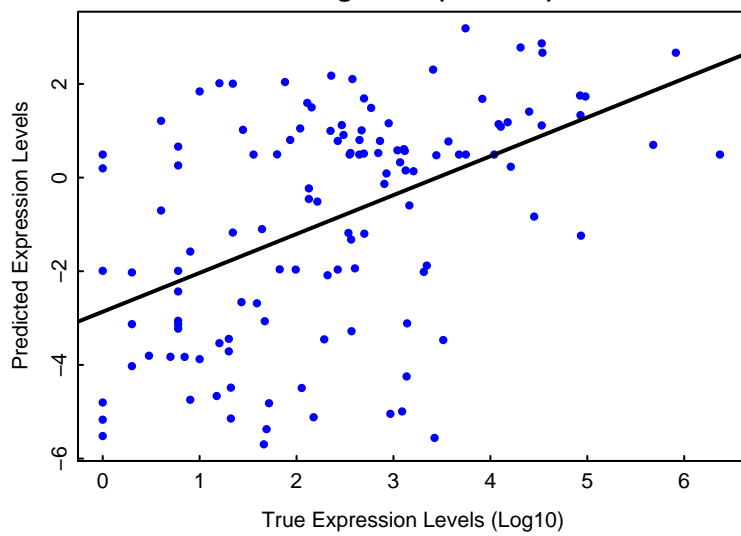

Supplement: Additional file 14 — Stage specificity of chromatin models for microRNA expression predictions. The chromatin model was trained using the chromatin and expression data of protein-coding genes at the EEMB stage. The model was then used to predict microRNA expression levels at six stages. R indicates the Pearson correlation coefficient between the predicted expression levels and the actual expression levels from RNA-seq experiments. [file gb-2011-12-2-r15-S14.PDF]
