# Supplementary material for: A statistical framework for modeling gene expression using chromatin features and application to modENCODE datasets
Source: Genome Biol. 2011 Feb 16;12(2):R15. doi: 10.1186/gb-2011-12-2-r15 (PMC3188797; doi:10.1186/gb-2011-12-2-r15)
Supplement: Additional file 11 — Supplementary documents about the Bayesian network analysis and so on. The file contains additional information about the Bayesian network analysis. [file gb-2011-12-2-r15-S11.PDF]

Supplemental documents:

Chromatin features form a complex high-order relationship network

Our analyses of the interactions between the above chromatin features only considered binary interactions between two features. For higher-order relationships involving more features, it is infeasible to perform the same type of analyses, as the number of feature combinations would become intractable. Also, the above analyses only suggest which features interact with each other, but do not explain how the features interact. In particular, the complex correlations between features and gene expression make it difficult to extract directional relationships between them (Additional file 10, Supplemental Figure S10A).

Bayesian network inference, as demonstrated in two recent studies [1,2]provides an effective solution to this issue owing to its desirable features, namely (1) it "explains away" indirect correlations and results in a network with the most probable interactions, and (2) it infers the most probable causality directions of interactions in the network based on the observed data and certain assumptions [1]. We therefore used Bayesian networks to study the higher order relationships between the chromatin features and gene expression.

We calculated the signals of the 26-chromatin features in the DNA region centered at the TSS (-1kb~1kb) of 27,310 transcripts. These features include 11 histone (H3 and HTZ-1) occupancy or histone modifications, and the binding of 8 factors involved in X-chromosome inactivation, 5 transcription factors, RNA polymerase II and the centromere protein-A homolog HCP-3. The signal profiles for all these chromatin features were measured by ChIP-chip or ChIP-seq at embryonic stage. Based on these data, we performed Bayesian network analysis to infer the directional relationship among features and gene expression. The confidence of the inferred relationships was estimated by using a bootstrapping approach [3] (See Methods). Supplemental Figure S10B shows the inferred relationships with confidence scores of at least 80%. As shown in the figure, many indirect relationships (Additional file 10, Supplemental Figure S10A) have been excluded from the network. For example, in the inferred network, there is only one histone modification, namely H3K4me3 that directly interacts with gene expression with high confidence, though many other features are highly correlated with gene expression. In other words, gene expression levels can be largely determined by the signals of H3K4me3, and the other chromatin features only indirectly affect gene expression by affecting the state of H3K4me3. Moreover, the inferred network correctly predicts some known interactions, such as the dependence of SDC-3 on SDC-2 for genomic binding [4]. Finally, the network also provides us with novel hypotheses about interactions among chromatin features for further experimental validation.

Detecting combinatorial effects using mutual information

Quantifying combinatorial regulation between different histone modifications via the linear models described assume *a priori* linear dependence. Mutual information measures the general dependence between two variables, and therefore serves as a complementary way to study interactions between histone

modifications (see methods). By classifying expression levels and different histone signals into Boolean variables (High/Low), we examined whether the mutual information between expression (E) and various logical combinations of pairwise histone signals (e.g. $I(E, H_1 \wedge H_2)$, $I(E, H_1 \vee H_2)$) is higher than the mutual information between expression and individual histone signals, i.e. $I(E, H_1)$, $I(E, H_2)$. We found that for feature pairs such as H3K36me3 and H3K4me2, the "AND" operation between them indeed gave a higher mutual information than the mutual information of the two individual features, meaning that the pair operates to a certain extent in an "AND" manner (Additional file 9, Supplemental Figure S9).

Methods:

Bayesian network analysis of chromatin features and gene expression

A total of 26 chromatin features and expression levels were considered for Bayesian network analysis. The binding profiles of these chromatin features were measured by ChIP-chip or ChIP-seq experiments and expression levels were measured by RNA-seq. All these data were collected from *C. elegans* samples at embryonic stage (EEMB or mixture embryo). For each chromatin feature, we calculated the weighted average of its signal in the DNA region centered at the TSS of each transcript. Specifically, the weighted average of feature i in transcript j is computed by $X_{i,j} = \frac{1}{2000}\sum_{k=1}^{2000} S_{i,j,k} w_{i,k}$, where $S_{i,j,k}$ is its signal at position k (the bases from -1kb~1kn around the TSS of each transcript is considered) and $w_{i,k}$ is the weight of feature i at position k. The weight for a chromatin feature at each position was determined based on its average density around the TSS region across all transcripts. In combination with the expression levels of all transcripts, we finally obtained a matrix X with 27 columns (26 features and expression) and 26,959 rows (transcripts).

We then discretized each column of matrix X by setting the top 5% as 1s and the remaining 95% as 0s. The resulted binary matrix was used for Bayesian network inference using Banjo 2.0 (http://www.cs.duke.edu/~amink/software/banjo/). Parameter settings were determined according to a previous study [1].

We applied a bootstrapping approach to estimate the confidence of inferred interactions by the Bayesian network analysis. Specifically, we computed 1,000 networks using Banjo, each based on a re-sampled matrix by randomly sampling rows with replacement. For an inferred interaction, the confidence score was denoted as the frequency of occurrence of the corresponding directed edge in the 1,000 Banjo networks. We finally output the inferred network model that contains all the interactions with confidence scores of at least 80%.

Detecting combinatorial effects using mutual information

We classified expression levels and different histone signals into Boolean variables (High/Low). The expression level of a gene is defined as high (low) if the value is larger (smaller) than the median, while histone signals were divided based on the same cut-off as Supplemental Figure S10A. Various logical combinations between histone features were determined using the Boolean vectors, and mutual information between sets of features and expression were calculated.

1. van Steensel B, Braunschweig U, Filion GJ, Chen M, van Bemmel JG, et al. (2010) Bayesian network analysis of targeting interactions in chromatin. Genome Res 20: 190-200.
2. Yu H, Zhu S, Zhou B, Xue H, Han JD (2008) Inferring causal relationships among different histone modifications and gene expression. Genome Res 18: 1314-1324.
3. Pe'er D (2005) Bayesian network analysis of signaling networks: a primer. Sci STKE 2005: pl4.
4. Ercan S, Lieb JD (2009) C. elegans dosage compensation: a window into mechanisms of domain-scale gene regulation. Chromosome Res 17: 215-227.
